# Bright Innovations: Review of Next-Generation Advances in Scintillator Engineering

**DOI:** 10.1021/acsnano.3c12381

**Published:** 2024-05-23

**Authors:** Pallavi Singh, Georgy Dosovitskiy, Yehonadav Bekenstein

**Affiliations:** † Solid State Institute, Technion-Israel Institute of Technology, Haifa 32000, Israel; ‡ Department of Materials Science and Engineering, Technion-Israel Institute of Technology, Haifa 32000, Israel; § The Nancy and Stephen Grand Technion Energy Program, Technion-Israel Institute of Technology, 32000 Haifa, Israel

**Keywords:** scintillator, photonic crystal, light output, nanocrystal, metascintillator, Purcell enhancement, coincidence time resolution, radioluminescence and medical imaging, perovskite nanocrystals

## Abstract

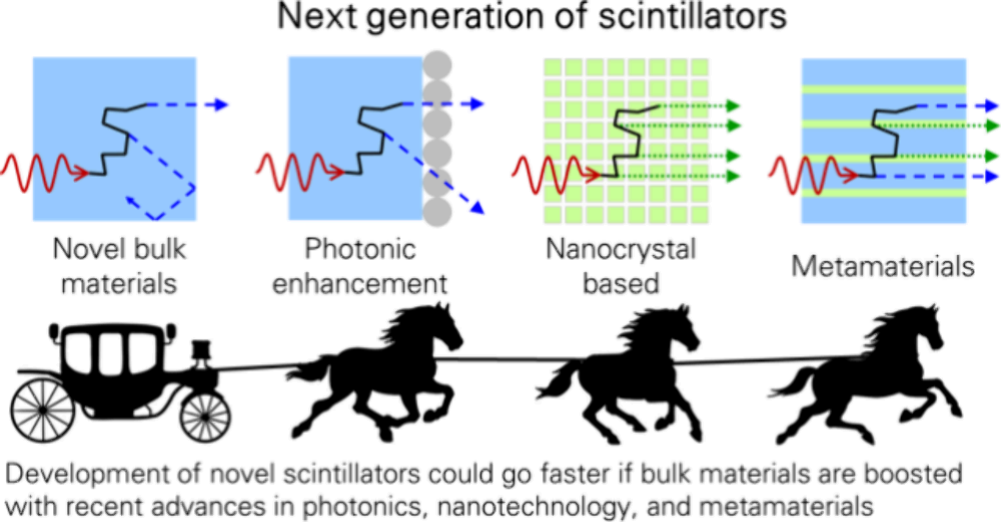

This review focuses on modern scintillators, the heart of ionizing radiation detection with applications in medical diagnostics, homeland security, research, and other areas. The conventional method to improve their characteristics, such as light output and timing properties, consists of improving in material composition and doping, *etc.,* which are intrinsic to the material. On the contrary, we review recent advancements in cutting-edge approaches to shape scintillator characteristics via photonic and metamaterial engineering, which are extrinsic and introduce controlled inhomogeneity in the scintillator’s surface or volume. The methods to be discussed include improved light out-coupling using photonic crystal (PhC) coating, dielectric architecture modification producing the Purcell effect, and meta-materials engineering based on energy sharing. These approaches help to break traditional bulk scintillators’ limitations, e.g., to deal with poor light extraction efficiency from the material due to a typically large refractive index mismatch or improve timing performance compared to bulk materials. In the Outlook section, modern physical phenomena are discussed and suggested as the basis for the next generations of scintillation-based detectors and technology, followed by a brief discussion on cost-effective fabrication techniques that could be scalable.

Scintillators are materials that emit visible light when interacting with high-energy ionizing radiation such as X-rays or γ-quanta (from <5 keV to >1 MeV; as generally accepted, we use the term X-rays for the radiation generated by a tube or a synchrotron and γ-quanta for the radiation of nuclear reaction origin, though physically they are the same) or high-energy particles such as energetic electrons, protons, neutrons, *etc*. These visible light photons are typically converted into electrical signals using a photoelectric effect when coupled with photodetectors, such as photomultiplier tubes (PMT), photodiodes, SiPMs, or charge-coupled devices (CCD).

Scintillation detectors are the core of radiation detection applications in medical imaging, e.g., X-ray radiography, Computed Tomography (CT), Single-Photon Emission Computed Tomography (SPECT), and Positron Emission Tomography (PET) scanners, homeland security, such as luggage and cargo screening at transport hubs, in nondestructive diagnostics and nuclear safety monitoring, as detectors for high energy physics used in colliders, as well as several other applications.^1^

Scintillation involves a sequence of processes of charge carrier generation, thermalization, transfer, and relaxation via luminescence. The evolution of photonic scintillators has been significant, transitioning from early 20th-century polycrystalline ZnS:Ag and CaWO_4_ materials, which remain in use today, to an extensive array of powder, ceramic, single crystalline, amorphous, and composite materials. The historical development of these materials is thoroughly documented in the subsequent references provided.^2,3^ Further, physical state of scintillators span from inorganic crystalline compounds (e.g., oxides such as garnets,^4^ oxide perovskites, ortho^5,6^ and pyro silicates, and halides, e.g., alkali metal halides,^7^ lanthanide halides, and perovskite halides^8^ and oxysulfides, *etc*.) to organic solids (such as organic crystals and plastics^9,10^), liquids,^11−13^ including liquid gases.^14^

Significant efforts are invested in improving the performance of scintillators, optimizing the photodetector, and efficiently implementing modern readout and signal processing electronics.^15^ A plethora of scholarly efforts are directed toward enhancing the performance metrics of scintillators, specifically in terms of light yield, rise time, and decay kinetics. This enhancement is achieved through sophisticated chemical composition engineering, along with strategic doping and codoping techniques. An example of this advanced approach is the systematic engineering of the intrinsic properties observed in garnet scintillators.^4,16−19^ Recent efforts in scintillators development are summed up in the review by Wang et al.^20^ However, in this mini-review, we will mainly focus on physical effects and performance improvement, achieved by patterning/modifying the scintillator’s surface or volume extrinsically with micro- or nanostructures without changing the chemical composition. The general concept of structured scintillators, as opposed to homogeneous ones, is reviewed by Lin et al.,^21^ and here we focus specifically on the materials with photonic and/or metamaterial properties. First, we discuss photonic structure to improve light extraction from scintillators to provide better photon transport to a photodetector without affecting their generation inside the scintillator. Further scintillator performance improvement by additional fast photons generation using the concept of meta-scintillators with energy sharing is considered, particularly highlighting halide perovskite quantum dots as a promising fast emitter. Then, works on the Purcell effect to increase and accelerate light emission are reviewed. Lastly, an outlook section highlights recently explored modern physical phenomena, which could be the basis for the next generations of photonic scintillators and further suggests the development of the described approaches by reviewing scalable and cost-effective fabrication techniques.

Further, to understand the subsequent discussion, we encourage the reader to visit the vocabulary section, where we briefly describe the main properties of scintillators, such as light output and other intrinsic properties (*e.g.,* stopping power, rise and decay time), which influence the output performance, such as CTR, count rate, energy resolution, and spatial resolution. For more details, please refer to the dedicated literature, *e.g.,* Lecoq et al.^3^ To provide the reader with a comprehensive understanding of typical scintillation parameter values, Table 1 compiles metrics for widely used scintillators across various domains. Additionally, it includes data for the less prevalent scintillators, which are specifically addressed within this review.

**Table 1:** Density, Light Yield, and Decay Time of Scintillators

scintillator	density (g/cm^3^)	light yield (photon/MeV @RT)^a^	decay time (ns)	refs
Bi_4_Ge_3_O_12_ (BGO)	7.13	8900 ± 450	300	ref (22)
				
Lu_2_SiO_5_:Ce (LSO)	7.4	30000	40	ref (23)
				
(Lu,Y)_2_SiO_5_:Ce (LYSO)	7.1–7.4	29000	35–40	ref (23)
				
CsI	4.51	16800	10	ref (3)
				
CsI:Na		38500	630	
				
CsI:Tl		51800	1000	
				
NaI:Tl	3.67	43000	230	ref (24)
				
BaF_2_	4.89	9950	0.87/0.88 (Fast)	ref (22)
			630 (Slow)	
				
Y_3_Al_5_O_12_:Ce (YAG:Ce)	4.57	30000	70	ref (25)
				
Gd_3_Al_2_Ga_3_O_12_:Ce (GAGG:Ce)	6.6	27900–49500	50–150	ref (23)
				
PbWO_4_ (PWO)	8.28	100	6	ref (26)
				
MAPbI_3_ single crystal	4	<1000	8 (Fast)	ref (27)
			425 (Slow)	
				
MAPbBr_3_ single crystal	3.8	<1000	4 (Fast)	ref (27)
			24 (Slow)	
				
CsPbBr_3_ single crystal	4.55	<1000	9 (Fast)	ref (28)
			64 (Slow)	
				
CsPbBr_3_ nanocrystal	4.55	6000–64000	<1–10	ref (29)
				
((C_4_H_9_NH_3_)_2_PbBr_4_	2.73	40,000	1.1, 8 (X-ray)	ref (30)
				
Cs_2_Ag_0.6_Na_0.4_In_0.85_Bi_0.15_Cl_6_		39,000 ± 7000	1 (Fast)	ref (31)
			2800 (Slow)	

a The references showing the characteristic values are cited here; however, multiple sources report different values for some materials. Their analysis was outside of the scope of the current review.

The preferred scintillators for PET (one of the most demanding commercial applications) are single crystalline Bismuth Germanate (BGO), Ce-doped Lutetium Oxy-Orthosilicate (LSO:Ce), and Lutetium Yttrium Oxy-Orthosilicate (LYSO:Ce), and therefore, they are extensively used for extrinsic modification studies. BGO crystals are affordable thanks to a relatively low melting temperature of 1050 °C, with a density of 7.13 g/cm^3^, and a light yield of 9000 ph/MeV; however, they suffer from a relatively long decay time (300 ns). L(Y)SO has a density of 7.1–7.4 g/cm^3^, a light yield of 25000–33000 ph/MeV, and fast decay (35–40 ns); nevertheless, its adoption is tempered by the relatively high costs associated with its crystal growth at elevated temperatures (above 2050 °C) and the expense of the raw materials required. Scintillators such as CsI:Tl, YAG:Ce, and the more recently developed GAGG:Ce are extensively utilized for a broad range of applications due to their general-purpose utility. PbWO_4_ is deployed in substantial volumes within high energy physics (HEP) detectors, whereas BaF_2_ garners attention for specialized applications, chiefly attributed to its rapid emission properties. Halide perovskites are recognized as burgeoning scintillator materials, a topic that will be explored in greater detail in a subsequent section of this review.

## Light Outcoupling in Bulk Scintillators Using Photonic Crystals

### Losses due to Total Internal Reflection in Traditional Bulk Scintillators

The typical scintillator materials are made of high-Z elements and have *high density*, as this is beneficial for effective stopping, especially toward high-energy photons (γ-quanta). The opposite side is their high refractive index (*n* = 1.8–2.15), leading to a mismatch between a scintillator and an out-coupling medium (mostly air with *n* = 1) between the scintillator and a detector. The refractive index mismatch limits the amount of extracted visible photons due to total internal reflection (TIR). Internally refracted photons keep traveling inside the bulk with an increased probability of reabsorption, reducing the extent of photons that can reach a photodetector and hampering an overall detector performance.

Scintillation light is considered to be emitted isotropically at a full 4π solid angle inside a scintillator. According to Snell’s law, a scintillated photon can effectively come out of the material and reach a photodetector only if the angle of incidence (*θ*_i_) is smaller than the critical angle (*θ*_i_ < *θ*_c_); all light incident at an angle greater than the critical angle will undergo TIR (Figure 2(A-i); e.g., LYSO, with a refractive index of 1.82, has a light output of only 8% at the crystal-air interface. Thus, improving photon transport from a scintillator to a photodetector bears significant potential to boost a detector performance. Refractive index mismatch can be reduced by applying a higher refractive index optical grease/fluid between the scintillator and the detector (e.g., PMT/APD), which allows more effective gathering of the reflected photons, and light output increases up to 50–60%;^32^ e.g., Singh et al. demonstrated LYSO (*n* = 1.82) coupled to a PMT detector using optical grease with *n* = 1.52 and *θ*_c_ 55.5° enabling light output up to 50%.^33^ Despite advancements in the field, the attenuation of light remains a substantial concern. Furthermore, the implementation of this methodology for remote readout via digital cameras presents considerable challenges. This limitation is particularly evident across the diverse spectrum of scintillation imaging applications, ranging from large-area medical/security applications to specialized screens intended for X-ray microscopy.

### Bioinspired Nanophotonic Scintillator: Borrowing from the Natural World

Diving into nature’s playbook, scientists have marveled at the natural world’s mastery of light and color: butterfly wings that dazzle without dye, peacock feathers that shimmer without a pigment, and fish that gleam thanks to the architecture of their scales. Inspired by these organic wonders, researchers have crafted artificial photonic crystals, enabling them to manipulate light in unique ways. These synthetic marvels are the building blocks for a current generation of nanophotonic devices, from waveguides to communication systems, proving once again that nature’s designs have set the stage for human innovation.^34^

**Figure 1:**
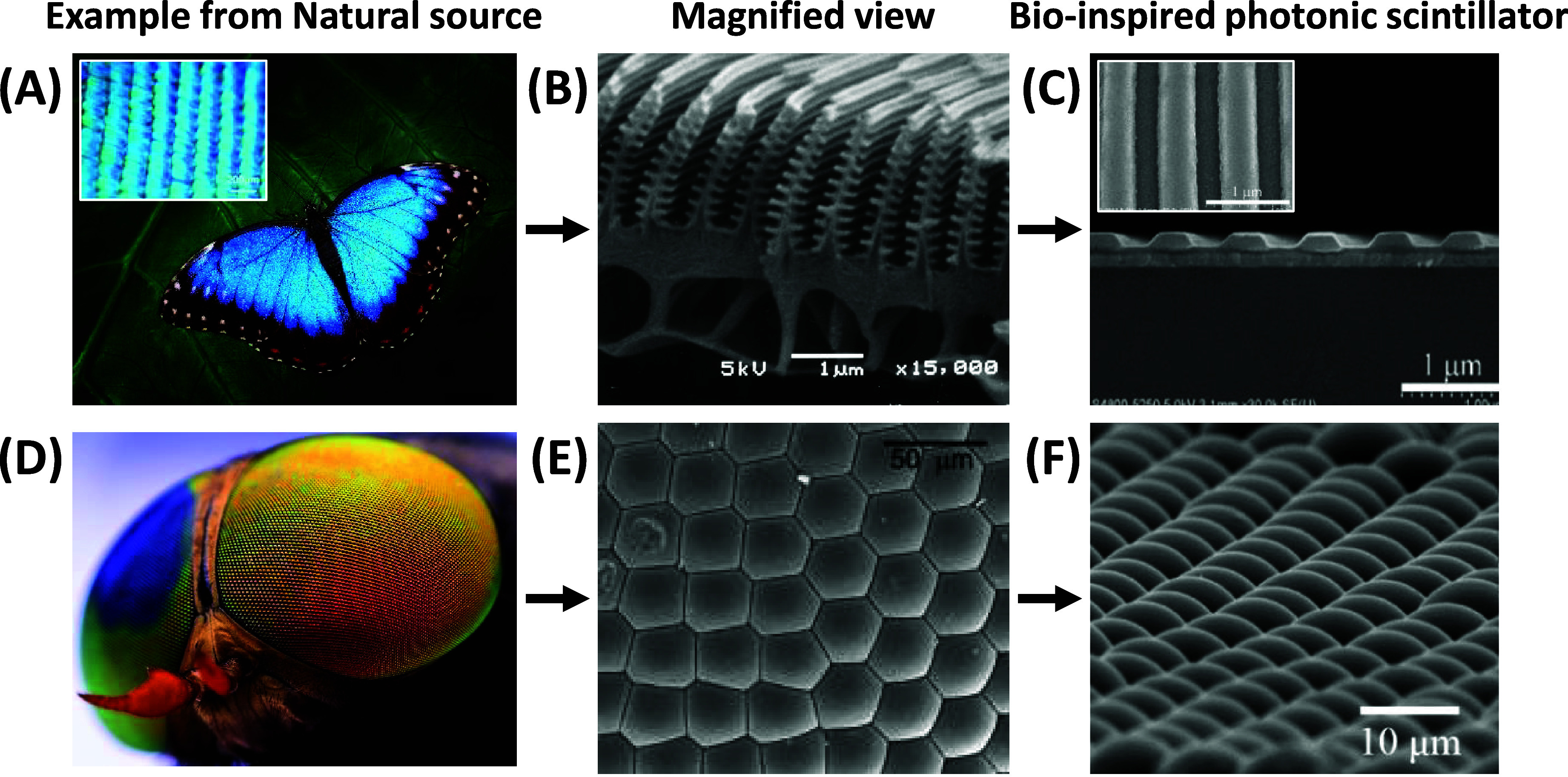
(A) The iridescence (dorsal side) of the butterfly wings originates from the microscopic array (licensed from Shutterstock.com), inset: wing showing scales under an optical microscope^35^ (reprinted with permission from ref (35). Copyright 2012 John Wiley and Sons). (B) Cross-sectional SEM image of the scale showing individual ridges (reproduced by the courtesy of Prof. Shinya Yoshioka, Tokyo University of Science). (C) Patterned lead halide perovskite film for optoelectronic applications:^41^ Top view (inset) showing analogy with butterfly wing pattern (reprinted with permission from ref (41). Copyright 2017 WILEY-VCH Verlag GmbH & Co. KGaA, Weinheim). (D) The insect’s compound eye shows thousands of tiny hexagonal ommatidia (licensed from Shutterstock.com). (E) SEM image of the ommatidia^42^ (Adapted with permission from ref (42). Copyright 2006 Oxford University Press). (F) SEM images of the microlens array on the top of LYSO bulk scintillator (lens diameter 4.5 μm), inspired by an array of ommatidia^43^ (Adapted with permission from ref (43). Copyright 2020 American Chemical Society).

1. Inspired by the iridescence, vibrant colors without pigments, but merely due to the nanoscale periodic structures, researchers have created artificial nanophotonic crystals that can control and manipulate light in various ways.^35^ Particularly, this greatly contributed to the design of waveguides and light-conducting structures for use in nanophotonic circuits and communication devices (Figure 1A–C).
2. Similarly, by mimicking the functioning of an array of micrometer-sized hexagonally packed tiny lenses called ommatidia found in insect compound eyes, which offer wider-angle view, higher light sensitivity, and enhanced detection for excellent moving objects,^36,37^ researchers have developed arrays of microscopic optical elements, such as nanoantenna, nanoapertures, microlens arrays, or photonic crystals, which can be used to manipulate light (Figure 1D–F). These systems benefit various applications, such as better absorption of solar irradiance by solar panels, efficient emission in light-emitting devices,^38−40^ and other solutions to challenges in optical design, light management, and sensing.

**Figure 2:**
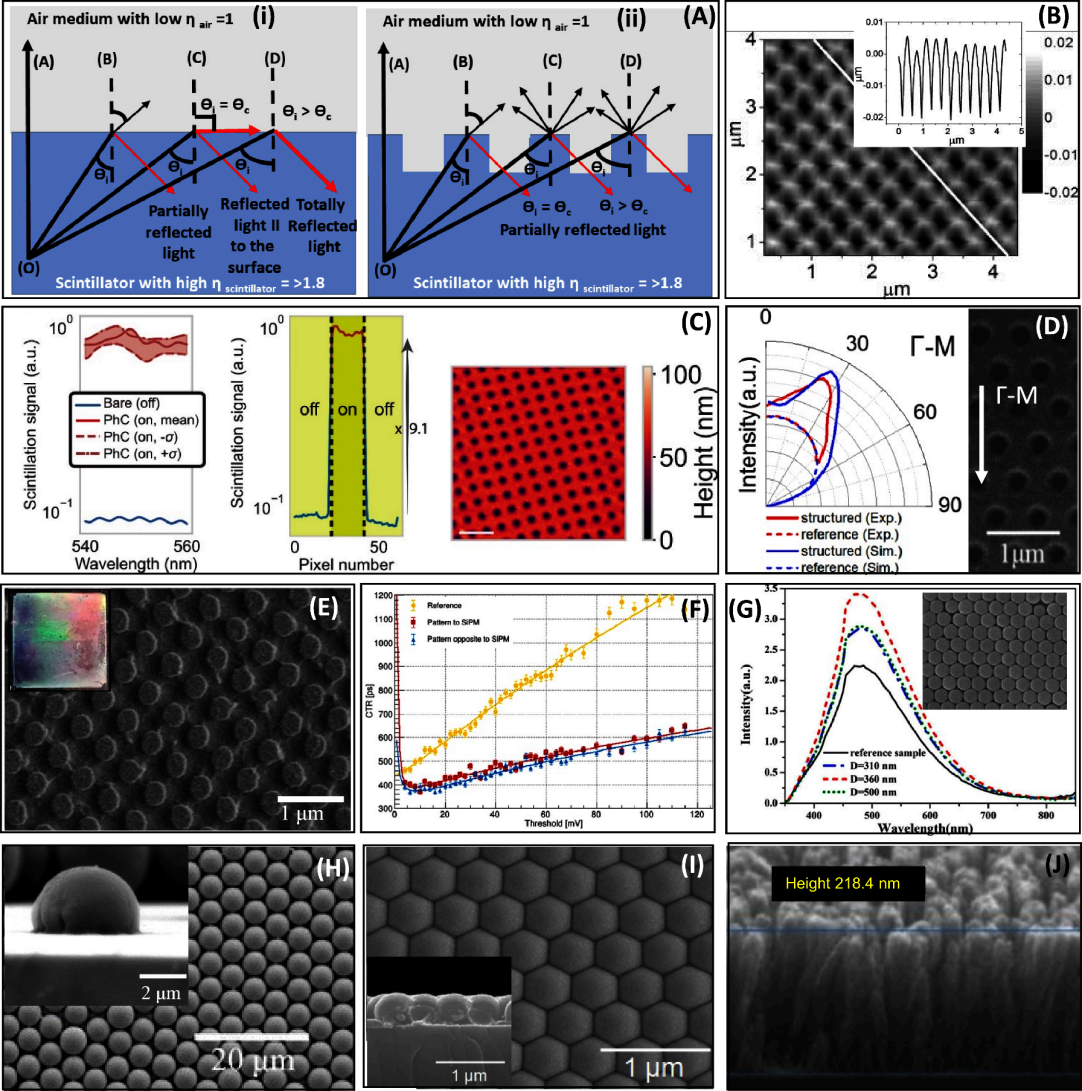
(A) Refraction and TIR of light from point O in a scintillator incident at different angles at the interface. θ_c_ and θ_i_ represent the critical angle and angle of incidence. (i) All photons hitting the crystal–air interface with an angle larger than θ_c_ undergo TIR. (ii) Extraction of a fraction of TIR photons with the PhC patterned interface. Here, the line thickness corresponds to the amount of light extracted and reflected. (B) Soft X-ray interference lithography: AFM of the patterned PMMA layer with TiO_2_ deposited on top^59^ (reprinted from ref (59) with permission of AIP Publishing). (C) Calculated scintillation spectrum of the YAG PhC. (Left) Integrated over the experimental angular aperture. (Middle) Measured scintillation along a line of the sample, including regions on (red) and off (blue) the PhC. (Right) AFM image of patterned YAG:Ce scintillator; scale bar–1 μm^65^ (from ref (65) reprinted with permission from American Association for the Advancement of Science). (D) Hot embossing: (right) structured polystyrene sample. (Left) Experimental and simulated angular profiles of light emission at the wavelength of 393 nm along Γ-M orientation^66^ (reprinted with permission from ref (66). Copyright Optica Publishing Group). (E) Nanoimprinting: square pillars of TiO_2_ on LYSO:Ce (inset: photograph) and (F) its CTR without Teflon wrapping or optical coupling^67^ (reprinted with permission from ref (67). Copyright 2019 The authors are under Creative Commons CC BY license). (G) Size-dependent light output of the BGO crystals coated with SiO_2_ nanospheres under UV excitation^68^ (reprinted from ref (68). with the permission of AIP publishing). (H) Multilens array formed from a self-assembled layer of partially melted polystyrene microspheres on LYSO:Ce surface (inset: single hemispherical microlens)^43^ (reprinted with permission from ref (43). Copyright 2020 American Chemical Society). (I) Self-assembled periodic arrays of polystyrene spheres conformally on LYSO coated with TiO_2_ layer (inset: cross section)^57^ (reprinted with permission from ref (57). Copyright The Optical Society). (J) Electron Beam evaporation: Vertical columnar structure of TiO_2_ on BGO with graded-refractive index^69^ (reprinted with permission from ref (69). Copyright 2020 AIP Publishing).

Inspired by the above, in the last few decades, micro- or nanostructuring has shown great potential in the field of scintillation in the form of PhC on the scintillator’s surface to further reduce the TIR and enhance the light output up to several fold, thus increasing the performance of scintillators and overall ionizing radiation detection efficiency (Figure 2A). The Outlook section also discusses some bioinspired ideas relevant to scintillators’ development.

In the past, research and development of scintillators was driven principally by experimental efforts. With the rapid improvement in computational capabilities, the role of simulation and modeling in scintillator research has been increasingly prominent. The use of DFT^44^ modeling in scintillator development is actively applied for the selection of candidate materials and properties^45^ and their performance optimization.^46−48^ Recently, even molecular dynamics simulation, originally intended for studying the physical movements of atoms and molecules, was used to improve fluoroaluminosilicate scintillation glass ceramics by modeling phase separation processes.^49^

Numerical modeling is widely used to develop scintillation detectors; GEANT4 (GEometry ANd Tracking) is one of the most widely used simulation software packages in particle and nuclear physics, accelerator design, space engineering, and medical physics. It is an open-source Monte Carlo simulation toolkit designed for simulating the passage of particles, including photons of different energies, through matter and their interaction with the PhC surface up to the photodetector. The reader can refer to the attached references for detailed information.^50−52^ We use numerical simulation results in this review along with the experimental ones.

### Research Results on Photonically Enhanced Scintillators

Experimental studies involve PhC coatings comprising thin layers of periodic submicrometer structures (such as nanospheres, nanocubes, square pillars, and patterned square/cylindrical air holes, *etc*.) of dielectric material (e.g., TiO_2_, SiO_2_, ZrO_2,_ etc.) deposited on the scintillator out-coupling surface with the periodicity of the order of the wavelength of the visible light (note: the periodicity of PhC can occur in two or three dimensions as well). It was shown by simulation^53,54^ that the evanescent field near the scintillator interface could couple to the nanostructures of the dielectric layer, forming the whispering gallery modes that can propagate in the plane of the layer of nanostructures as a dielectric waveguide due to the coupling among these adjacent nanostructures. The light in the guided modes could be diffracted into the far field by the periodic structure as leaky modes, making light extraction possible beyond the critical angle thus increasing out-coupled light.

This approach was supported by various experiments showing improved light output, listed in Table 2 and a few reports mentioning improvement in temporal or spatial resolution. Diffraction (and, hence, light extraction) properties of PhC gratings depend on factors such as height (*h*), lattice constant (*a*), diameter of the nanostructure (*d*), real and imaginary parts of the refraction index of the PhC material, and filling factor. Table 2 is categorized based on various fabrication techniques, showing improvement in light output using different shapes and sizes of PhC (most works involve simulation studies followed by experimental investigations). Different units are used in the literature to report the light enhancement, so we purposefully give only the relative improvements. Gain in the measured light signal is reported in the articles cited here, and we have used the terminology “light output” for it. The following observations could be made based on Table 2:

(a) The significant variation of light output was seen even with the similar combinations of a scintillator and a dielectric PhC, having different dimensions of the nanostructure, e.g.,
  (i) The light output of LYSO coated with TiO_2_ PhC increases by 53%^55^ and up to 70%^56^ in the 2D pattern of air holes of different sizes engraved on TiO_2_.
  (ii) The light output of LYSO coated with a monolayer of polystyrene nanospheres varies from 38% to 220%, depending on the size of the nanospheres.^43,57^
(b) PhCs with higher RI materials lead to stronger scattering amplitudes, therefore, more light extraction and brighter diffraction orders, e.g., gain in light output by 50% and 40% and energy resolution by 9% and 3.6% when GYGAG nanoimprinted with polymers with RI 1.875 and 1.825, respectively.^33^
(c) Similarly, the deposition of an additional layer of a higher refractive index, e.g., TiO_2_, is an additional way to fine-tune a photonic layer,^58^ e.g., light output increases by 33% and 101.6% in BGO/PMMA (*n* = 1.5) and BGO/PMMA (*n* = 1.5)/TiO_2_ (*n* = 2.5) scintillator.^59^
(d) Light extraction by the photonic layer is more efficient for plate-shaped scintillators,^43,57,60−64^ which is probably caused by more photons hitting the escape surface; thus pattering allows reduced scintillator thickness keeping the light output, thus improving spatial resolution in imaging applications.

**Table 2:** Improvement in Light Output of Scintillators Using PhC of Different Dielectric Materials and Dimensions Based on Various Fabrication Techniques^a^

S. no.	scintillator and dimension of PhC	relative light output improvement
**Simulation Only**		
**1**	LYSO, LuAG, BGO, LuAP (1.3 × 2.6 × 8 mm^3^)	Light output increased by nearly 100%.
	Cylindrical air holes etched on Si_3_N_4_ (*d =* ∼170–250 nm, *h* = 400 nm, and *a* = 300–430 nm)^70^	
		
**2**	BaF_2_, GaN, ZnO scintillators	Self-collimation efficiency improvement in PhC by reducing beam width (μm) after 1 mm propagation in a media.
	PhC structure of cylindrical air holes^71^	BaF_2_ 94.5 μm → 2.8 μm
		GaN 539.6 μm → 4.8 μm
		ZnO 402.2 μm → 4.1 μm
	
**Soft X-ray Interference Lithography**		
**3**	BGO (1 × 10 × 20 mm^3^)	
	(a) Conical holes in PMMA by soft-X-ray interference lithography (labeled as sample A) (*d* = ∼300 nm, *h* = 45 nm, and *a* = 400 nm)	(a) Light output increases by 33% in BGO/PMMA (*n* = 1.5).
	(b) sample A is followed by a TiO_2_ layer deposition by ALD^59^ (Figure 2B)	(b) Light output increases by 101.6% in BGO/PMMA (*n* = 1.5)/TiO_2_ (*n* = 2.5).
		
**4**	YAG:Ce crystal (⌀25.4 mm × 0.2 mm)	Light output increased by ∼180% in the normal direction.
	The square lattice of air holes (d = ∼380 nm, *h* = ∼200 nm *a* = 600 nm)^62^	
		
**Electron Beam Lithography**		
**5**	LSO (1.2 × 2.6 × 5 mm^3^)	Light output increased by 20–60%.
	Various 2D-PhC on the Si_3_N_4_ (*d* = ∼170–500 nm, *h* = 45 nm, and *a* = 280–640 nm)^72^	Angular distribution gain varied from 0.98 to 1.57.
		
**6**	GAGG:Ce crystal (10 × 10 × 0.1 mm^3^)	Light output increased by 43% with optical grease coupling (*n* = 1.465).
	The square lattice of air holes etched on TiO_2_ (*d* = ∼360 nm, *h* = 226 nm, and *a* = 528 nm)^73^	
		
**7**	LYSO (2.0 × 2.0 × 5.0 mm^3^)	Light output increased by 53% and energy resolution by 43.8%.
	2D-square periodic array of air holes engraved in TiO_2_ deposited on LYSO (*d* = ∼300 nm, *h* = ∼152 nm, and *a* = 450 nm^55^	
		
**Focused Ion Beam**		
**8**	(a) LYSO (0.8 × 0.8 × 10 mm^3^)	(a) Light output increased by 30% in PhC engraved on LYSO.
	Square matrix of holes engraved directly on the surface (*d* = ∼320 nm, *h* = ∼300 nm, and *a* = 500 nm)	
	(b) LYSO (1.4 × 1.4 × 7.2 mm^3^)^56^	(b) Light output increased by 70–80% depending on the holes’ depth, diameter, and lattice parameter.
	Square matrix of holes engraved on TiO_2_ deposited over scintillator (*d*: = ∼200 nm, *h*: = ∼300 nm, and *a* = 500 nm).^56^	
		
**9**	YAG: Ce (20 μm thick)	Light output increased up to 9-fold.
	Square lattice patterned on area 215 × 215 μm (*a* = 430 nm)^65^ (Figure 2C)	
		
**Nanoimprint Lithography**		
**10**	Polystyrene based plastic scintillator (20 × 20 × 1 mm^3^)	Light output increased by 64% at 25° emergence angle along Γ-M orientation and 58% at 20° emergence angle along Γ-K orientation. Overall, the angle and wavelength-integrated light output increased by 45%.
	Array of cylindrical holes (*d* = 300 nm, and *a* = 600 nm)^66^ (Figure 2D)	
		
**11**	LYSO:Ce (10 × 10 × 10 mm^3^)	Light output and energy resolution improved by 50% and 10%, respectively.
	Nanopillars of TiO_2_ (*d* = ∼320 nm, *h* =∼300 nm, and *a* = 580 nm)^67^ (Figure 2E,F)	CTR improves from 450 ps → 390 ps.
		
**12**	LYSO:Ce crystal (1 × 1 × 1 cm^3^)	Light output increased by 68% (measured with PMT) and 140% (measured by SiPM) for the unwrapped air-coupled crystal and <5% for the Teflon wrapped, glycerol coupled one (*n* = 1.47 at 420 nm).
	The square-packed pyramidal pattern on UV-curable polymer (*n* = 1.82) (*d* = 880 nm, and *h* = 1275 nm)^74^	CTR improves from 535 to 315 ps under γ-quanta excitation.
		
**13**	LSO:Ce film^75^	Light extraction enhancement, wavelength-integrated, by 106–110% at the emergence angles of 52–56° under UV excitation.
	PMMA film patterned with nanoimprinting and coated with TiO_2_ using ALD	
	Triangular pillar (*a* = 600 nm, *h* = 300 nm, and *d* = 400 nm)	
		
**14**	(a) LYSO (1 cm^3^)	Light output increased by 20% and ER by 18% for LYSO.
	(b) GYGAG (⌀10 mm × 5 mm)	Light output increased by 50% and ER by 3.6% for GYGAG.
	(c) SrI_2_:Eu (⌀30 mm) with a quartz window	Light output increased by 42% and ER by 15% for SrI_2_:Eu.
	Hexagonally close-packed polymer nanocones with *n* = 1.825^33^	All—Teflon wrapped and with optical grease (*n* = 1.52) coupled, under γ-quanta excitation
		
**15**	CdSe/ZnS QDs	
	CdSe/ZnS QDs spin-coated on TiO_2_-covered triangular lattice of PMMA nanoimprinted on a quartz substrate (*a* = 600 nm, *h* = 310 nm, and *d* = 400 nm)^76^	Light output increased by 200% in the normal direction in wavelength-integrated emission spectrum (both under UV and X-ray excitation).
		
**Self-Assembled Monolayer**		
**16**	BGO (1.0 × 10.0 × 20.0 mm^3^)	Light output increased by 45.3% using 360 nm nanosphere under UV excitation.
	SiO_2_ nanospheres (*d* = 310, 360, and 500 nm)^68^ (Figure 2G)	
		
**17**	BGO crystal (3 × 10 × 20 mm^3^)	Increase in wavelength- and angle-integrated light output by 72% with UV excitation and by 68% (estimation) with γ-quanta excitation, no crystal wrapping.
	Polystyrene spheres (*d* = 500 nm)^77^	
		
**18**	BGO crystal (20 × 10 × 1 mm^3^)	The light output was enhanced by 115% and 150% at 0° and 35°, respectively, under UV excitation; under X-ray excitation in the normal direction, the results are close.
	Mixed-scale microstructures fabricated by the multistep process:	
	(1) polystyrene nanospheres (400–600 nm) self-assembly	
	(2) ALD of a TiO_2_ shell	
	(3) polystyrene microspheres (4.5 μm) self-assembly; heat treatment to melt the microspheres^78^	
		
**19**	LYSO (1 × 10 × 20 mm^3^)	Light output increased by 25% (less wavelength and angular dependence) under UV excitation.
	Anodized aluminum oxide (AAO)	
	Average interpore distance = 350–450 nm, *h* = 750 nm^79^	
		
**20**	LYSO:Ce (⌀12 mm × 25 mm)	Light output increased by 34–40%. CTR improvement: 892 to 725 ps in PhC (18.7% rise), optical coupling to photodetector is not specified under γ-quanta excitation.
	TiO_2_-coated polystyrene nanosphere (*d* = 500 nm)^80^	
		
**21**	LYSO:Ce crystal (3 × 10 × 20 mm^3^)	Light output increased by 38% under UV excitation.
	A self-assembled monolayer of polystyrene spheres with diameters 300–620 nm^81^	
		
**22**	LYSO crystal (10 × 20 × 1 mm^3^)	Light output increased by 226% at θ_em_ = 45°, by 152% — angle-integrated under UV excitation; under X-ray excitation in a 45° direction, the results are close.
	A monolayer of polystyrene microspheres (*D* = 1–4.5 μm), heat-treated to partially melt and form hemispheres^43^ (Figure 2H)	
		
**23**	LYSO (1 × 10 × 20 mm^3^)	Wavelength and angle-integrated light output increased by 149% under UV excitation, and about 200% in the normal direction under X-ray excitation.
	A monolayer of polystyrene nanospheres (*d* = 414 nm) coated with a TiO_2_ layer with thicknesses of 30, 58.8, and 90 nm^57^ (Figure 2I)	
		
**24**	β-Ga_2_O_3_ (5 × 5 × 1–3 mm^3^)	1 mm—NSAs are better
	Polystyrene nanosphere (NSAs, *d* = 400 nm) and Nanocone array (NCAs = 180 nm)^60^	2 mm—NSAs and NCAs have similar performance
		3 mm—NCAs are better (light output increase by 42%)
		All—under X-ray excitation.
		
**25**	CsI:Na crystal (⌀30 mm × 2 mm)	Light output increased in the normal direction, wavelength-integrated, by 179% under X-ray excitation.
	TiO_2_-coated monolayer of polystyrene nanospheres (*d* = 300–600 nm)^63^	
		
**26**	EJ-212 plastic scintillator (⌀30 mm × 0.2 mm)	Light extraction enhancement, wavelength- and angle-integrated, by 120% under X-ray excitation, by 135% under UV.
	A monolayer of polystyrene nanospheres (*d* = 500 nm)^64^	
		
**27**	Tb^3+^-doped silicate glass (3 × 5 × 10 mm^3^)	Light output increased by 25% at 45° under X-ray excitation.
	A monolayer of polystyrene nanospheres (*d* = 200–600 nm)^82^	
**Electron Beam Evaporation**		
**28**	BGO (11 × 13 × 2 mm^3^)	Light output increased by 53% (without grease) and 29% with grease under γ-quanta excitation; crystals were covered with reflective paint.
	Vertical columnar structure of ZrO_2_ with graded refractive index (*n* = 1.3 to 2.0)^83^	
		
**29**	BGO (20 × 20 × 1.5 mm^3^)	Light output increased by 15% under γ-quanta excitation, and reflective wrapping and no optical grease were used.
	Vertical columnar structure of TiO_2_ with graded refractive index (*n* = 1.15–2.17)^69^ (Figure 2J)	

a Abbreviations used for PhC dimension are in parentheses, e.g., hole diameter (*d*), depth (*h*), and period (*a*). The scintillator-photodetector coupling medium is specified if other than air. The type of excitation source is specified. The information on reflection wrapping is given where available. The reader is encouraged to access the original publications for further technical details.

Applying a photonic pattern to a scintillator’s surface is a universal approach to improving light output; therefore, it may benefit detector performance in all applications, depending on this parameter, i.e., literally all scintillator applications.

## Enhancement of Time Resolution by a Meta-Scintillation Approach

**Figure 3:**
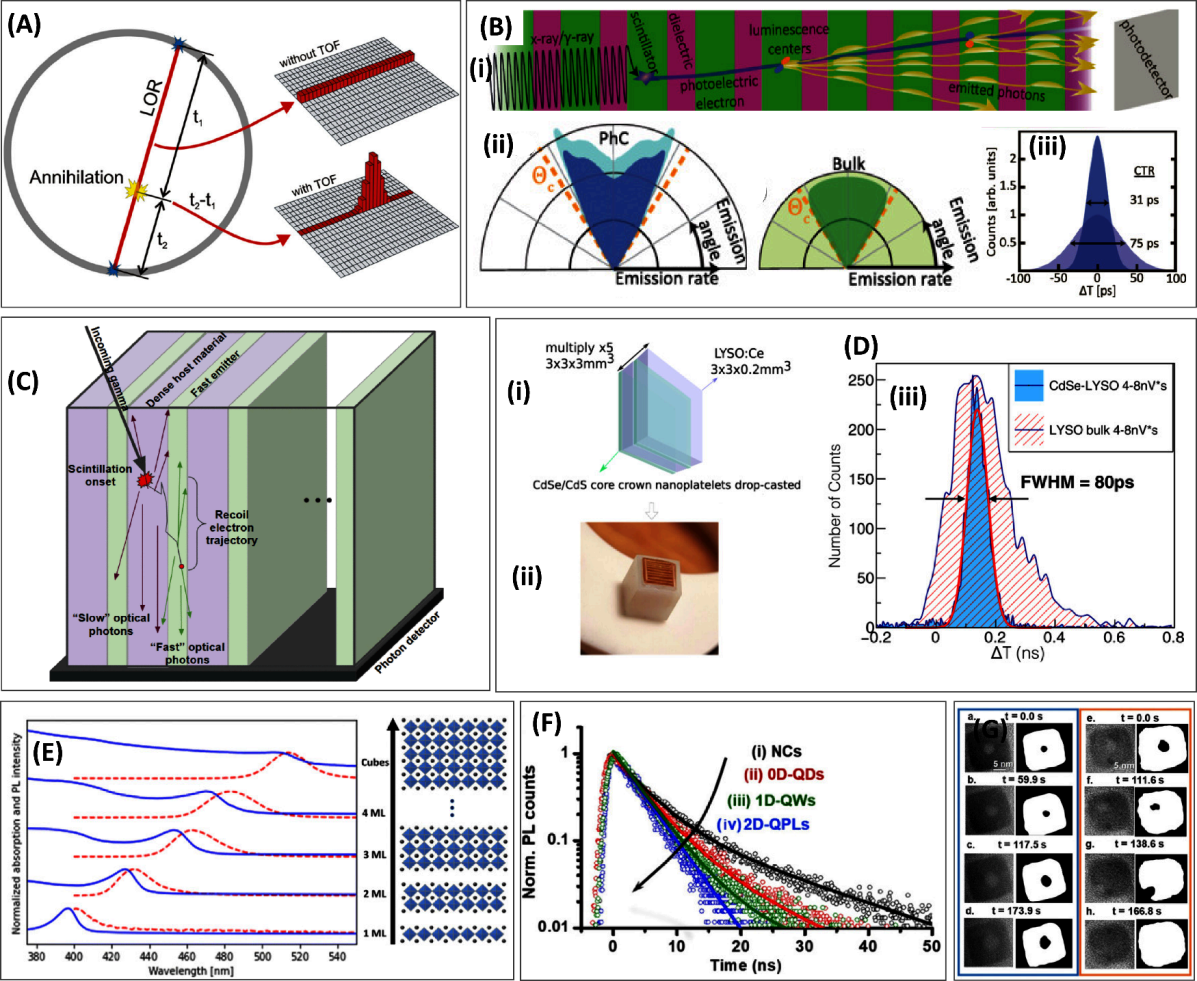
(A) Principle of time-of-flight PET scanner^98^ (under Creative Commons CC BY 4.0 license). (B) (i) Schematic showing scintillation in a photonic crystal scintillator based on Purcell enhancement. (ii) The total emission rate for the PhC (turquoise) and the outcoupled part (blue) and bulk (light green) and the outcoupled part (green) as a function of emission angle. The PhC effective emission rate enhancement is the ratio between the blue area in PhC and the green area in bulk. (iii) Improvement in CTR from 75 ps in bulk to 31 ps in PhC structure^87^ (reprinted with permission from ref (87) Copyright 2020 by the American Physical Society). (C) Schematics of a meta-scintillator topology: alternate thin slices of a dense host material and a fast emitter are placed next to each other, allowing energy sharing and scintillation^99^ (under Creative Commons CC BY 4.0 license). (D) (i) and (ii) Schematics and CdSe/CdS nanoplatelets + LYSO sampling pixel. Delay time distribution of events reduced to 80 ns in CdSe-LYSO meta-scintillator compared to bulk LYSO (charge integration between 4 and 8 nVs)^100^ (under Creative Commons CC BY 4.0 license). (E) Absorption and emission spectra illustrating Stokes shift changes for CsPbBr_3_ nanoplatelets of different thicknesses, here ML stands for monolayer^101^ (reprinted with permission from ref (101). Copyright 2022 the Royal Society of Chemistry). (F) Photoluminescence decay curves (excitation 365 nm) for CsPbBr_3_ perovskites in the form of (i) nanocrystals, (ii) quantum dots, (iii) quantum wires, (iv) quantum platelets^102^ (reprinted with permission from ref (102). Copyright 2021 American Chemical Society). (G) HR-TEM images of before (left) and after plasma treatment (right) of Cs_2_AgInCl_6_ nanocube and their binary images showing void defect dynamics when exposed to different accumulated electron doses^103^ (reprinted with permission from ref (103). Copyright 2021 Wiley-VCH GmbH).

The above examples of photonic modification lead to better light output, and, therefore energy and temporal resolution; however, there is demand for further improvement in temporal resolution for high-energy physics experiments, and inspired by them are functional imaging techniques such as PET.^2^ PET scans are crucial for early-stage diagnostics, which detects lesions before any apparent change in anatomy. It shows real-time metabolic activity in tissues and organs using even picomolar (pM) levels of radiotracers, unlike other medical imaging modalities such as CT scans and MRI, which use mM and μM, respectively. In PET scanning, a radiotracer is injected into a patient, comprising a biologically active molecule, such as glucose, with a radioisotope attached, which can decay through the β^+^ mechanism, emitting a positron. The lesion cells absorb more radiotracer than the healthy ones as they require more energy for their fast and uncontrolled growth. These accumulated radiotracers do not metabolize and constantly release positrons. Annihilation occurs when a positron meets an electron, producing two oppositely directed (180°) 511 keV γ-quanta (Figure 3A). The PET scanner, equipped with scintillator detectors in a 360° configuration, detects these γ-quanta, which allows for determining a line of response (LOR), which indicates that e^–^e^+^ annihilation followed by γ-quanta emission occurred somewhere along the LOR. The high temporal resolution of detectors allows measuring the precise place of the γ-quanta emission along the LOR using time-of-flight (TOF) information, leading to improved statistical properties and better signal-to-noise ratio of the image (Figure 3A).^84^ In other words, TOF adds longitudinal information to the traditional line of response, allowing more precise localization of the annihilation process (Figure 3A).

An important figure of merit of a PET detector is coincidence time resolution [(CTR) – time resolution of a pair of opposite detectors in measuring 511 keV annihilation γ-quanta], along with the stopping power and cost. The lower the CTR, the lesser the imprecision; e.g., a CTR of 500 ps corresponds to an imprecision zone of 7.5 cm, which can be further reduced to 3 and 1.5 cm if the CTR can be reduced to 200 and 100 ps, respectively. Currently, the best reported TOF-CTR values are nearly 205 ps, demonstrated in the commercial device Biograph Vision 450,^85^ which allows for the uncertainty of 30 mm along LOR. The extreme challenge is to reduce this value to 10 ps (uncertainty of 1.5 mm), which improves the current image reconstruction procedure and even allows imaging via direct capturing of events without reconstruction.^15^ This reduces the radioactive doses without lowering the image quality, which allows the broader use of PET for various screening types and patient groups.

Temporal resolution depends not only on the scintillator but also on the photodetector and the read-out electronics. Achieving a CTR of 100 ps is already challenging and has a noticeable positive effect. Metamaterials are proposed as one of the approaches to break through the limitations of classical bulk scintillators^86^ and achieve lower CTR. We review two concepts of metamaterials. One employs the Purcell effect to accelerate luminescence,^87,88^ and the other—the production of prompt photons,^84^ even in small numbers (few hundred), along with a main scintillation pulse, either by adding a bulk or nanocrystalline fast-emitting scintillator.

### Enhancement of Light out-Coupling Based on the Purcell Effect

Properly designed PhCs can improve light emission characteristics due to the Purcell effect of the scintillator. Only a few theoretical reports are available, listed in Table 3. A theoretical study by Kurman et al.^87^ suggests a layered periodic structure of scintillator and dielectric spacer materials, whereby by tuning their thickness and refractive indexes, one can engineer the photonic band structure and thus control the photonic local density of states for each light frequency and propagation angle (Figure 3(B-i)). This effect increases with the difference in the refractive index of the two alternating layers. Mainly, the angular distribution of the emitted light can be shaped by enhancing the scintillator’s emission into detectable directions, simultaneously suppressing the emission in other directions, which otherwise will be lost due to TIR, and the spontaneous emission rate can be increased (Figure 3(B-ii)).^87−89^ The simulation showed a potential 5-fold increase in detectable photons in the 1D PhC of LYSO compared to bulk LYSO, along with an improved emission rate in the former. The calculated enhancement of CTR by a factor of 2.4 in PhC shows the potential of photonic scintillators in fast time-of-flight detectors (Figure 3(B-iii)).^87^ The effect is sensitive to the thickness uniformity of LYSO layers: a standard deviation of 6–7 nm for an 800-layer structure (total thickness of ∼220 μm) destroys the effect. Furthermore, it was shown that a multilayer structure with varying thicknesses for different layers, given their thicknesses are accurately optimized, could also be efficient Purcell effect structures, potentially improving spatial resolution in imaging applications.^90^ Bizarri et al.^91^ suggest using another side of the Purcell effect to shift a scintillator’s emission spectrum to better match the photodetector’s quantum efficiency, which can give up to a 2-fold increase in a photodetector output signal. Lately, Ye et al.^92^ experimentally showed the nanoplasmonic Purcell effect in (BA)_2_PbBr_4_ perovskite thin film deposited on top of HfO_2_-covered Au layer. The nanoplasmonic scintillator showed an improved PL and RL signal and faster decay compared to the reference ((BA)_2_PbBr_4_ thin film on glass, as well as a 182% enhancement in modulation transfer function at the spatial frequency of 4 line pairs per mm.

**Table 3:** Theoretical and Experimental Reports Showing Time Improvement in Various Metascintillators^a^

	meta-scintillators	lifetime and CTR (fwhm)
**Enhancement of Light Out-Coupling Based on the Purcell Effect**		
**1**	†LYSO and GOS	A 5-fold increase of detectable photons in case of LYSO PhC and CTR improvement (75 ps → 31 ps).
	1D periodic layered PhC structure of above scintillators with SiO_2_^87^	∼3.5-fold increase of detectable photons in case of Gd_2_O_2_S:Tb PhC.
		
**2**	†CsI:Na	∼200% increase in the photodetector signal.
	CsI:Na sandwiched between 1D external PhC cavity made of alternating layers of TiO_2_ (*n* ≈ 2.87) and porous SiO_2_ (*n* ≈ 1.06)^91^	
		
**3**	#(BA)_2_PbBr_4_	The best results are for 45 nm scintillator film: 120% enhancement in RL signal intensity.
	Layered structure: (BA)_2_PbBr_4_ (45–496 nm)/ HfO_2_ (15 nm)/ Au (70 nm)/ glass^92^	(BA)_2_PbBr_4_:
		τ_PL1_ 1.0 ± 0.1 ns, τ_PL2_3.9 ± 0.4 ns
		τ_RL1_ 1.5 ± 0.2 ns, τ_RL2_ 4.1 ± 0.4 ns, τ_RL3_ 7.7 ± 0.8 ns
		(BA)_2_PbBr_4_/HfO_2_/Au:
		τ_PL1_ 0.7 ± 0.1 ns, τ_PL2_ 2.9 ± 0.3 ns
		τ_RL1_ 0.7 ± 0.1 ns, τ_RL2_ 1.4 ± 0.2 ns, τ_RL3_ 3.4 ± 0.3 ns
		
**Meta-Scintillators Based on the Alternating Layers of High-Density Scintillators and Fast-Emission Scintillators**		
**4**	†Alternating layers of LYSO with EJ-232 and BaF_2_^97^	Bulk LYSO: CTR 200 ps (3 × 3 × 20 mm^3^)
		LYSO-BaF_2_: CTR 133–173 ps (3 × 3 × 17–19 mm^3^)
		LYSO-EJ232: CTR 141–177 ps (3 × 3 × 21–25 mm^3^)
		
**5**	†Alternating layers of BGO and EJ232^126^	Bulk BGO: CTR 276 ps
		BGO (100 μm)- EJ232 (100 μm): CTR 204 ± 49 ps (3.0 × 3.1 × 15 mm^3^)
		BGO (100 μm)- EJ232 (50 μm): CTR 220 ± 41 ps (3.0 × 3.1 × 15 mm^3^)
		
**6**	†Plates of BGO, LYSO 0.3 × 25.5 mm^2^ and plates of plastic EJ232, EJ232Q 0.1 × 3.1 mm^2^ assembled in the structure of 15 or 24 mm height, read out from the side.^127^	CTRs with timewalk correction:
		BGO-EJ232: 225 ps (15 mm)
		LYSO:Ce-EJ232Q: 107 ps (15 mm)
		BGO-EJ232: 253 ps (24 mm)
		LYSO:Ce-EJ232Q: 120 ps (24 mm)
		
**7**	#BGO, ∼100 μm	Bulk BGO: CTR 271 ± 14 ps
	EJ232, ∼100–200 μm^95^	BGO/100 μm EJ232: CTR 239 ± 12 ps (3 × 3 × 15 mm^3^ pixel)
		BGO/200 μm EJ232: CTR 197 ± 10 ps (3 × 3 × 15 mm^3^ pixel)
		
**8**	#BGO with EJ-232 and BaF_2_^97^	Bulk BGO: CTR 400 ps 3 × 3 × 15 mm^3^
		BGO/BaF_2_: CTR 241 ps
		BGO/EJ232: CTR 205 ps
		
**9**	#BGO, 3 × 3 × 1 mm plate, in which a 5 × 5 array of 400 μm diameter channels is drilled.	Bulk BGO: CTR 185 ± 6 ps
	((BA)_2_PbBr_4_) is grown inside the channels.^128^	BGO-(BA)_2_PbBr_4_: CTR 168 ± 5 ps
		Measured in coincidence with LSO:Ce:0.4%Ca with nominal CTR 62 ± 3 ps
		
**10**	#BGO 300 μm/BaF_2_ 300 μm^129^	Bulk BGO: CTR 535 ps 3 × 3 × 15 mm^3^
		BGO/BaF_2_: CTR 241 ps (108.5 ps for fast events subset with the highest energy sharing)
		
**Meta-Scintillator Based on Nanocrystal and Bulk Scintillator**		
**11**	#CdSe/CdS nanoplatelet films on LYSO (3 × 3 × 0.2 mm^3^)^100^ (Figure 3 (D (i) and (ii))	Bulk LYSO: CTR 180 ps
		LYSO-CdSe: CTR 80 ps (10 plates assembly, 3.8 × 3.8 × 3 mm^3^) (Figure 3 (D-iii))
		
**12**	#CsPbBr_3_ thin films on GGAG:Ce^130^	Shorter rise time in meta-scintillator:
		Bulk GGAG:Ce - 8 ns
		CsPbBr_3_ on GGAG:Ce - 30 (dynamic) and 50 ps (static) depending on the deposition
		Shorter decay time in meta-scintillator:
		GGAG:Ce (200 ns; 63%, 660 ns; 37%, 80 ps; 1%)
		GGAG:Ce + CsPbBr_3_ (700 ps, 1%, long; 98%, 120 ps, 3%)

a †Simulation. #Experimental.

### Meta-Scintillators Based on a Combination of High-Density Scintillators and Fast-Emission Scintillators

Another possible meta-structure for improved temporal resolution involves the combination of high-Z scintillating material with good stopping power and fast optical photon emitters. The possible design would be alternating periodic layers of the above two. If their thicknesses are small enough, γ-quanta absorption could mainly take place in a high-Z material, and the recoil electrons will still experience substantial interaction with the fast emitter while crossing through it, depositing energy there, which is called energy sharing (Figure 3C). This architecture enables significant improvements in timing resolution by producing fast photons on top of the standard scintillation pulse. In some cases, it may also allow determination of the 3D localization of the gamma interaction point, thus providing depth of interaction capability—another detection regime desired for PET scanners to correct for possible parallax error.

The first experimental study used combinations of ∼200 μm thick plates of BGO and LYSO (PET detector materials with good stopping power) and ∼250 μm thick plates of BC-422 fast organic plastic scintillators in a 3 × 3 × 3 mm pixel.^93^ It was shown that some detection events indicate the sought-for energy sharing between the two components, and these events could be separated. Modeling of stopping power and energy sharing allows formulation guidelines for engineering metamaterial scintillators with improved timing.^94^ In the following, more refined experiment, plates of BGO (100 μm) and EJ232 plastic (100–200 μm, parameters similar to BC-422) were combined. A significant improvement over the CTR of bulk BGO was demonstrated; CTR of 197 ps for 3 × 3 × 15 mm pixel of 100 μm BGO/200 μm EJ232 Vs 271 ps for bulk BGO (Table 3).^95^ It should be noted that smaller pixels yield better CTR due to less inhomogeneity in the light collection.

BC-422 and EJ-232 improve CTR because of their fast emission properties but at the cost of stopping power due to lower Z, which is unfavorable for PET applications even with improved time resolution. A low fraction of energy-sharing events is another consequence. BaF_2_ crystals have fast scintillation components with subnanosecond decay time, emitting photons at 190–220 nm, and a much higher stopping power than plastics; the recent developments in SiPM technology allow their detection with 22% efficiency.

It was shown by simulation that combinations of BGO and LYSO scintillator with fast BaF_2_ crystal have a good compromise between the number of shared events detected and the maximization of the average fast photons.^96^ CTR values of 241 and 205 ps for BGO/BaF_2_ and BGO/EJ-232 were measured experimentally compared to 400 ps for bulk BGO.^97^ This study shows that the CTR of 200 ps (the CTR of current LYSO-based Biograph Vision PET) can be achieved even with BGO, which is 3 times less expensive than LYSO. Similarly, the bulk LYSO has measured CTR of 200 ps, further decreasing by 30–70 ps in LYSO/BaF_2_ and LYSO/EJ-232 meta-scintillators according to modeling in the same study.

Table 3 summarizes the examples of metascintillators with improved performance.^97^ As the determined CTR values depend on measurement and data processing details, subjects of separate discussions, CTR values for the reference samples are provided.

### Metascintillator Based on Fast Emitting Nanocrystal and Bulk Scintillator

Heterostructures combining standard bulk scintillators (such as LSO, LYSO, BGO) with bright nanocrystal scintillators, e.g., CdSe^100,104^ or lead halide perovskites, e.g., CsPbBr_3_,^105,106^ have a potential to make the next step to reach CTR of 10 ps. In nanocrystals, quantum confinement of excitons greatly increases the probability for radiative recombination due to increased spatial overlap between the electron and hole, improving quantum yield and accelerating emission. CdSe quantum dots are well-known for high light yield and fast emission, which makes them a reasonable candidate for hybrid scintillators.^104,107^

Halide compounds are well-known hosts for scintillator materials, and recently halides with the perovskite structure, particularly lead halide perovskite nanocrystals, have gained much attention in the past decade^108^ because of certain advantages over chalcogenide quantum dots, making them potential next-generation scintillators^109^ and semiconductor radiation detectors.^110^ In addition to tunable band gap and good light yield, they contain high-Z elements (e.g., Cs, Pb, and Br/I), require inexpensive raw materials, possess solution processability, and require easy growth conditions, which can make their upscaling possible. Lead halide perovskites, and in particular CsPbBr_3_, show giant oscillator strength and photoluminescence lifetimes that are an order of magnitude shorter than typical semiconducting nanocrystals, promising for ultrafast scintillation and detection.^109^ Also, CsPbBr_3_ nanocrystals have bright tunable narrow emission^111,112^ and reach near-unity quantum yield^113^ without thick inorganic shelling, indicating defects tolerance, unlike traditional semiconducting nanocrystals.^114^ Unfortunately, these materials experience strong self-absorption due to their small Stokes shift, and recently our group published a report on how composition tuning in double perovskites allows regulating Stokes shift by moving absorption and/or emission bands.^115^ Moreover, at the nanoscale, and by the engineering of dimensionality of the nanoparticles, one can influence both the Stokes shift (Figure 3E) and the luminescence decay time (Figure 3F). This was demonstrated with CsPbBr_3_ nanoplates and their assemblies in planar waveguides.^116,117^

Recently, X-ray scintillation experiments of CsPbBr_3_ showed strong absorption and intense radioluminescence. Chen et al.^109^ reported ultrahigh sensitivity of lead halide perovskite NC-based X-ray scintillators with a detection limit of 13 nGy s^–1^, three orders lower than 5.5 μGy s^–1^, the typical X-ray dose used for medical imaging.

Recent work by Zaffalon et al.^118^ presented the γ-ray scintillation properties of nonfluorinated CsPbBr_3_ (∼1,500 photons/MeV) and fluorinated F:CsPbBr_3_ NCs with improved luminescence and significantly suppressed temperature quenching even at 373 K (∼8,500 photons/MeV, which is comparable to commercial BGO).^119^ These lead halide perovskite QDs exhibited exceptional radiation hardness for γ-radiation doses ranging from 1 kGy to 1 MGy, probably due to the crystal’s high defect tolerance and self-healing properties. Recently, Cahen et al. extensively discovered self-healing in bromide and iodide analogs of lead halide perovskite crystals and thin films by damaging the material with a high-intensity pulse laser (equivalent to 10^6^ suns).^120−123^ Recently, our group has demonstrated the possibility of affecting diffusive trajectories of voids in lead-free Cs_2_AgInCl_6_ nanocubes.^103^ Surfaces of nanocrystals passivated by organic molecules (called ligands) support the internal structural integrity of perovskite nanocrystals by means of confining void defects to their interior, whereas nonpassivated nanocrystals demonstrated self-healing by diffusion of the voids to the surface and further outside of the crystal (Figure 3G).^103^ The reader could refer to the review by Wibowo et al.^124^ for further reading on halide perovskites scintillator applications.

Despite significant interest in lead halide perovskites in radiation detection, only preliminary results are available on hybrid/metamaterial scintillators using them, and they are listed in Table 3; e.g., CsPbBr_3_ NCs were drop cast on a Lu_*x*_Y_2–x_SiO_5_:Ce wafer, and a fast component with sub-nanosecond decay was registered in both PL and RL kinetics.^125^

### Applications Based on Reviewed Enhancements

It is noteworthy to understand that a single scintillator cannot be ideal for all the applications, and the reader may refer to the following literature for comprehensive information on scintillator requirements for certain applications.^1,2,20,131,132^ Here we briefly name some applications that could benefit from the above-mentioned enhancements; *e.g.,* PET was mentioned above as an application that could benefit from enhanced light output, energy resolution, and timing. These parameters are relevant for other modalities of medical imaging as well; *e.g.,* very fast scintillators with high light output may make way to time-of-flight measurements in CT and X-ray radiography, allowing significantly reduced dose load on a patient.^133,134^ High-energy physics uses high-volume scintillator detectors for calorimetry (particle energy measurement); the sampling calorimeter concept, based on a combination of tiles of fibers of different materials in one detector (shashlik or spaghetti type, correspondingly), presents a prefiguration of energy sharing metamaterials.^135,136^ At the same time, detectors dedicated to measuring the precise timing of the studied events, particle tracking, or beam characterization utilize thin sensitive layers, so it is the available niche for nanophotonically enhanced materials, and both enhanced timing and light output would play positive roles in these applications.^2,137,138^ Other rapidly developing applications such as X-ray microtomography and microscopy, including synchrotron-based imaging, demand good spatial resolution, which can be achieved by a photonic approach.^139^ Other applications in the areas of security, nondestructive testing, scientific measurements, etc., may benefit from nanophotonic and metamaterials approaches when these underlying technologies are mature enough to allow for volume materials.

Aside from emission characteristics, some other scintillators’ properties are crucial for certain applications, e.g., maintaining light yield at elevated temperatures—for well logging, and low sensitivity to gamma-quanta—for neutron physics. High radiation hardness is a widely required characteristic, in high-energy physics, the nuclear industry, CT, radiography, and all high-flux applications. Usually, crystals with a rigid lattice are considered more radiation hard than compounds with a softer lattice; *e.g*. oxide crystals are less prone to radiation damage than halides: LYSO maintains 60% of its light output after 340 Mrad irradiation, BGO showed 35% after 200 Mrad, and CsI – 30% after only 1 Mrad.^140^ However, lead halide perovskites—promising components of nanophotonic scintillators—show surprisingly high radiation hardness; thin film of Cs_0.05_FA_0.81_MA_0.14_PbI_2.55_Br_0.45_ barely showed any drop-in power conversion efficiency after a 2.3 Mrad dose,^141^ and F passivated CsPbBr_3_ NCs of showed >70% PL quantum yield after 100 Mrad irradiation.^107^ One of the possible reasons is that radiation-induced defects do not have spectral overlap with the emitted light, as it happens in garnets, or self-healing, as seen in case of lead/lead-free halide perovskite.^103,123^ For further reading, we may refer to general works or focused articles on a specific material of interest.^142,143^

Further, moving from laboratory to commercial application, scalability and cost-effectiveness come into play, which is considered in the following outlook section.

## Outlook

### Improvement in Scintillation Performance Using Smart Materials

In this section we present the development directions for photonic and metamaterial scintillators, which we find to be the most promising. This paragraph contains our personal views derived from the existing literature.

**Figure 4:**
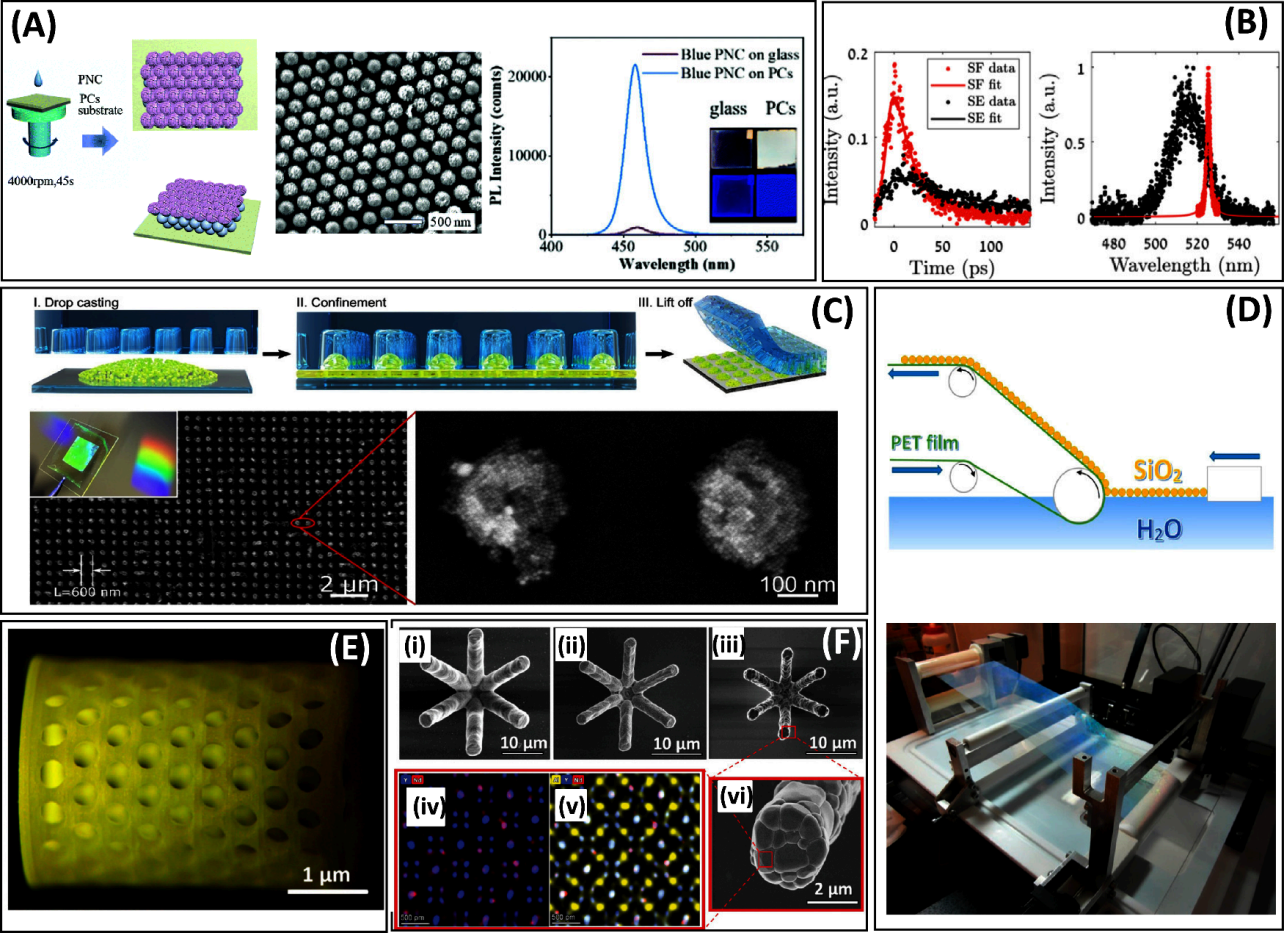
(A) Schematic of the preparation of CsPb(Br_0.51_Cl_0.49_)_3_ nanocrystals on SiO_2_ photonic crystal, SEM image of the resulting structure, and its typical emission spectra excited at 405 nm compared to the same nanocrystals on plain glass^147^ (reprinted with permission from ref (147). Copyright 2021 Royal Society of Chemistry). (B) Decay time and emission spectra of CsPbBr_3_ nanocrystals superlattice at 10 K excited by defocused electron beam (black curves), causing spontaneous emission, and focused electron beam, causing superfluorescence (red curves); red curves show a much narrower emission band and a faster decay time^153^ (reprinted with permission from ref (153). Copyright The Optical Society). (C) Schematics of patterning of the layer of 10 nm CsPbBr_3_ nanocrystals by nanoimprinting with a poly(dimethylsiloxane) template and SEM of the patterned layer; inset shows the sample photo under white light^178^ (under Creative Commons CC BY 4.0 license). (D) Schematics and photograph of a roll-to-roll Langmuir–Blodgett deposition of SiO_2_ spheres (550 nm) onto poly(ethylene terephthalate) film^179^ (reprinted with permission from ref (179). Copyright 2016 American Chemical Society). (E) Gd_3_Al_2_Ga_3_O_12_:Ce ceramic scintillator 3D-printed using DLP stereolithography^183^ (under Creative Commons CC BY 4.0 license). (F) YAG:Nd ceramic structures, fabricated using a two-photon printing, SEM after printing (i), after heating to 1100 °C (ii), after heating to 1500 °C (iii), (iv), and HRTEM of one of the crystals comprising the YAG structure at zone axis 100 with element image of Al, Y, and Nd (v), and of Y and Nd (vi)^184^ (reprinted with permission from ref (184). Copyright 2020 WILEY-VCH Verlag GmbH & Co. KGaA, Weinheim).

Several theoretical works were mentioned earlier in which scintillator emission is enhanced by photonic effects of 1D or 2D patterning of a material’s volume.^71,88,89^ A number of experimental works prove the potential of this approach, but from those, only a few reports involve radioluminescence enhancement, e.g., a patterned layer of CdSe/ZnS quantum dots shows 200% radioluminescence enhancement.^76^ However, more significant effects were demonstrated for PL enhancement rather than for scintillation, and a few examples are mentioned here such as ∼3-fold increase in luminescence was observed in the CH_3_NH_3_PbBr_3_ layer deposited onto a patterned substrate,^144^ a 23.5-fold increase in PL emission accompanied by a 7.9-fold reduction of spontaneous emission rate is reported for patterned continuous film of CsPbBr_2.75_I_0.25_,^145^ 20-fold fluorescence increase and more than 5-fold photoluminescence acceleration in CH_3_NH_3_PbBr_3_ photonic crystal film based on self-assembled 3D opal of PS spheres.^146^ Furthermore, colloidal halide perovskite nanocrystals are a perfect material for photonic enhancement; for example, colloidal crystals CsPbBr_0.51_Cl_0.49_ spin-coated onto 3D SiO_2_ have shown a significant rise in fluorescence intensity compared to the same nanocrystals on the plain glass along with a noticeable decrease in a lifetime from 6.20 to 2.60 ns (Figure 4A).^147^ We find it of great interest to study these effects systematically with radioluminescence excitation, as their potential was probably not fully uncovered for scintillation applications. The next step in continuation would be fabrication of 3D photonic scintillators, enhancing both emission, as described above, and transport of light, as shown here.^71^

Superfluorescence^148,149^ is of interest for application in scintillators due to the fantastic possibility to accelerate light emission dramatically. Particularly, superlattices of lead halide perovskite nanocrystals can emit in this modality with a decay time down to 14 ps at an excitation density of 1200 μJ cm^–2^;^150^ however, the studies are usually performed at liquid He temperature. Recently, room-temperature superfluorescence was demonstrated in (PEA)_0.4_CsPbBr_3_ thin films^151^ characterized by a light pulse fwhm of about 10 ps, and room-temperature upconverted superfluorescence was observed. In another study, Er@NaYF_4_:Yb@NaNdF_4_:Yb core–shell–shell nanoparticles showed room temperature superfluorescence with a decay time of 46 ns, which is 10,000-fold faster than normal up-conversion luminescence in this system, 455.8 μs.^152^ In our opinion, the possibility of enjoying this type of fast emission at room temperature is exciting for scintillation applications and, therefore, warrants further research. Recently, our group reported superfluorescence cathodoluminescence in CsPbBr_3_ superlattices using pulsed electron beam excitation to excite the superlattices, and the superfluorescent signal is characterized by a decay rate of 22.5 ps as opposed to spontaneous emission decay at 128 ps; thus superfluorescence has a potential to amply the time performance of scintillators by speeding up its decay (Figure 4B).^153^

Though they are not exactly nanophotonic, we mention other fast emission processes which are also considered for fast-timing applications, such as Cherenkov radiation, hot intraband luminescence, and cross-luminescence.^15^ Particularly, Cherenkov detectors are well-studied for high-energy physics applications and are now considered promising for TOF PET.^154,155^ A recent development in photodetectors and readout electronics allows getting time information even with 10–20 photon/511 keV, which makes a BGO potential combined Cherenkov-scintillation detector.^156−158^ An alternative energy sharing metascintillator design could use Cherenkov radiators as a high Z absorber and a “fast” component, which needs to be supplemented by some high light yield materials. We want to note that fabricating meta-scintillators with thinner layers would allow for a more reproducible event-to-event response, which raises a question of suitable fabrication techniques.

Further, lead halide perovskite-based scintillators are at an early research stage. Though self-absorption is addressed as one of the challenges for these materials, the sensitive layers are currently below ∼100 μm.^159−161^ Thicker layers are desirable to provide higher stopping power to γ-quanta in the energy range important for practical applications, but their performance will suffer from high self-absorption. One of the solutions to decrease self-absorption in thicker lead halide perovskite NCs layers may be in patterning them.^144,162^ Our hands-on experience with halide perovskites confirms that they are the ideal material for applications involving patterning. The other approach would be to modify the nanocrystals themselves, and modeling of quantum dots could be a great boost to the experimental work. However, a typical nanocrystal consists of thousands of atoms, and the simulation of such a system using the existing approaches is limited by the available computational power. In contrast, direct simulation of scintillation in nanocrystals is hard to come by. A Stokes shift is a parameter defining the self-absorption of luminescence material and, therefore, is of utmost importance for scintillators, which can be modeled; e.g., our group’s recent work helped uncover the mechanism of Stokes shift tuning in double perovskites.^115^ Brennan et al.^163^ used first-principles DFT modeling to reveal the mechanism of the Stokes shift size dependence in CsPbBr_3_ nanocrystals where crystals with sizes up to 4.4 nm were modeled. This allowed the overlap of the edges of the modeled and experimental data. Future improvements in computational power will greatly benefit this field, allowing the simulation of larger and more complex systems.

Another approach to increase the stopping power of nanocrystal-based detectors is to use heavy hosts. Here the idea that could be borrowed from natural objects could help, which is transparent nanostructured materials; e.g., careful dispersion and compaction of cellulose nanofibers (extracted from wood powder) with diameters of ∼15 nm give a transparent plastic-like material^164^ as all the inhomogeneities appear to be too small to cause light scattering. The same effect provides transparency of carefully dried SiO_2_ gels, while they remain composed of tiny particles. These materials look like suitable hosts for doping with temperature-sensitive species, which was demonstrated for silica xerogels both using organic dyes^165^ and nanoparticles.^166^ The next logical step is to widen the range of xerogels’ compositions to heavier compounds, particularly scintillating ones, and use fast and bright halide perovskites as doping nanoparticles.

Earlier the photonic crystal structures were extensively reviewed to improve light collection in scintillators. Here we want to mention another approach we find quite promising. Solution-processed hybrid perovskites were shown to have the potential as photodetectors.^167^ This facilitates the construction of a futuristic hybrid detector material, combining a scintillator with a photodetector with vast deposition and patterning versatility.^168^ Discussions with peers have shown us another direction in photodetection, which could yield creative ideas—bioinspired dyes, such as melanin, chlorophyll, and carotenoids. They possess tunable/broadband absorption,^169^ and quantum efficiency of the processes corresponding to photoabsorption in chlorophyll could reach unity under optimal conditions^170^ and the captured sunlight energy is transferred to reaction centers by dye combinations on a 10–100 ps time scale.^171^

### Appropriate Scalable Fabrication Techniques

As shown in the previous sections, significant improvement of the scintillation characteristics is possible *via* patterning materials on the submicro-/nanometer scale. Scalable fabrication of these materials using cost-effective techniques is vital for their commercialization and challenging; therefore, exploring suitable fabrication techniques is crucial for further developing photonically enhanced and metamaterial scintillators.

The methods for fabricating layered structures, such as those required for energy-sharing meta-scintillators or Purcell-enhanced layered scintillators, are well-known for decades and are being constantly improved and utilize layers deposition from liquid or vapor phases using chemical reactions or various sputtering techniques;^172,173^ therefore, they are not reviewed here. Rather thick layers of materials are needed to detect radiation of practically important energies, e.g., 2–3 mm thick layers of an appropriate scintillator for ∼60–150 keV γ-quanta (CT) and at least 10–20 mm thick for 511 keV γ-quanta (PET),^1,174^ Therefore, a high growth rate is a key consideration while selecting a technique capable of providing reasonable production costs.

Several techniques for applying patterns to flat surfaces (2D-ordered structures) are well-studied in semiconductor technology^175,176^ or laser technology.^177^ We will briefly explore the appropriateness and limitations of different techniques from the point of view of scalability. The photolithography approach is high throughput, capable of producing large samples, and is widely applied in industry; it requires an elaborate sequence of chemical processes, which complicates its use for an early R&D stage (nevertheless, it is used, but this approach becomes more attractive for large scale production). Nanoimprinting is also a widely applied, potentially less-expensive, and high-throughput technique; still, its selection is limited to those materials that could be soft enough to be imprinted (e.g., polymers or nanocrystal-based ink layers) (e.g., Figure 4C).^178^ Focused ion beam (FIB) processing is versatile and can be applied to a wide range of materials, but it is slow and expensive and thus is not applicable to produce either large sizes or large quantities of materials; contrary to this, laser ablation/direct laser writing offers the formation of 2D patterns with photonic properties faster and more affordable than with FIB; however, spatial resolution is still inferior.^177^ The method’s versatility allows its usage in all stages of the research and R&D process. Also, photonic crystals with large areas and relatively small thicknesses can be fabricated using the self-assembly approach,^179,180^ including potentially inexpensive and high-throughput roll-to-roll processes (Figure 4D). However, preparing photonic single crystals large in all three dimensions (without significantly misoriented blocks) is still a challenging task. Among the fabrication techniques, self-assembly looks particularly interesting due to the possibility of putting together colloid crystals of two types of particles, resembling ionic crystal lattices.^181,182^ It gives additional freedom for scintillator properties engineering for future sophisticated designs.

Combining halide perovskite nanocrystals, possessing a fast and bright emission, with heavy inorganic hosts, allows for achieving a high stopping power. Nanocrystal formation inside a host, well described in the relevant section of review by Lin et al.,^21^ is a known approach but still has the potential to be developed. The other way is to apply external densification; given the general thermal instability of halide perovskite nanocrystals, low-temperature densification procedures developed recently, such as cold sintering or hydrostatic consolidation, may be a solution.^185^ Additionally, these approaches may be used for an attempt to fabricate fully dense 3D-patterned material, e.g., by using the 3D-patterned green body, or a dense scaffold filled with NCs.

3D printing of scintillator materials, both organic and inorganic, with spatial details on the order of hundred microns, was demonstrated (Figure 4E), which is much larger than needed for photonic enhancement but may be enough for some types of metamaterials with energy sharing.^183,186,187^ 2-photon stereolithography is a commercially available technique, which allows high-resolution 3D-printing. It was used to fabricate objects from YAG, a popular scintillator host^116^ (Figure 4F), and photonic structures^140^ with submicrometer resolution. However, this method is still relatively expensive and slow. Further development of 3D printing will probably result in higher throughput techniques with the necessary capabilities.

Last but not least is a lately practiced strategy aimed at enhancing the efficacy of research methodologies, specifically through the integration of automated high-throughput synthesis techniques and data-driven investigations.^188^ Combinatorial search based on high-throughput synthesis was applied in scintillator development already more than 10 years ago.^189,190^ These works were precursors to the modern data-driven approaches. Recently, the data-driven approach allowed extraction of patterns of the Stokes changes from the data of 2000 samples of several types (nanoplatelets and nanocubes, produced either by manual or high-throughput automated synthesis).^101^ The modern trend in this direction is applying machine learning strategies,^191^ which are discussed in the dedicated section concerning scintillator development.^20^

## Conclusions

The vision of the present review is to bring the reader to the forefront of scintillator technology. In pursuit of this goal, we have curated and spotlighted pivotal research that demonstrates enhancements in scintillator light output through extrinsic modifications. The existing research concludes that better coupling of photonic nanostructures with scintillators using a range of (and, possibly, a combination of) dielectric materials with different dimensions can offer an improved light output. We also reviewed the works aimed at improving the temporal resolution of scintillators for better performance in the TOF-PET scanners and other fast-timing detectors using the meta-scintillator approach. We have brought our experience in radiation hardness and self-healing capability of lead/lead-free halide perovskite-based materials, which makes them a sustainable scintillator. In the outline section, we highlight approaches that could further improve scintillator performance based on the extensive discussion in the main part. We discussed the potential of nanocrystals as a fast and luminescent component in meta-material architecture for TOF-PET application and emphasized lacking scintillation studies. Lastly, we delineate prospective avenues for the refinement of fabrication methods that could revolutionize the extrinsic structuring and patterning of scintillators, facilitating their mass production for a multitude of practical implementations.

## Vocabulary: Brief Information on Scintillators’ Properties

Stopping power: the efficiency of the scintillator material to absorb the ionizing radiation; may be expressed, e.g., as an attenuation coefficient. It depends on the exact radiation type and its energy, determining the mechanism of interaction with matter.^192^ Scintillators with high density composed of elements with higher atomic numbers (*Z*) offer better-stopping power to γ-quanta and charged particles.
Light yield (LY): the number of emitted photons per absorbed energy with typical units of photons/MeV. It influences the essential properties of a scintillation detector—energy, time, and spatial resolution. This term could imply two meanings: (a) physical light yield, the number of photons generated by a material;^1^ (b) technical light yield, the number of photons that could be directed toward the photodetector and measured. The former is mostly an intrinsic material property, while the latter is influenced by the efficiency of light transport from inside of a scintillator to a photodetector and is deteriorated by effects such as self-absorption, scattering, and internal reflection. *For clarity, we call the technical light yield “light output” throughout this review (although in some literature these terms are used as synonyms).* Typical values for widely used scintillators are given in Table 1.
Rise time (τ_r_): the characteristic time, defining the leading edge of a scintillation pulse. It is shaped by dynamics of excitation transfer to emission levels, and for widely used scintillating materials, can span from the subpicosecond domain in self-activated scintillators such as PbWO_4_^193^ to several nanoseconds in activated scintillators, like GAGG:Ce.^194^
Decay time (τ_d_): the characteristic time (times) of exponential decay of one or (usually) more scintillation components. It defines the ability to discriminate between two consecutive scintillation pulses, thus limiting the detector count rate and contributing to detector time resolution. It depends on the kinetics of the processes of (1) charge carriers transport to emitting centers and (2) intracenter luminescence.
Temporal resolution (*R*_*t*_): the ability to precisely time-tag the moment of detection of radiation quanta. The scintillator time resolution depends on the scintillation pulse shape (the shorter the rise time and decay time, the better the resolution) and light yield (the more, the better) and is often approximated by eq 2; however, the actual dependence may vary significantly depending on a specific type of a scintillator.^195^ 2 The time resolution of two detectors in detecting simultaneous events is called a coincidence time resolution (CTR) and is a crucial parameter in time-of-flight PET scanners.
